# Confirmed organophosphorus and carbamate pesticide poisonings in South African wildlife (2009–2014)

**DOI:** 10.4102/jsava.v86i1.1329

**Published:** 2015-12-09

**Authors:** Christo J. Botha, Heleen Coetser, Leonie Labuschagne, Andre Basson

**Affiliations:** 1 Department of Paraclinical Sciences, University of Pretoria, South Africa; 2 Toxicology Laboratory, Agricultural Research Council-Onderstepoort Veterinary Institute, South Africa

## Abstract

During a six-year period (from January 2009 to December 2014), specimens collected from 344 cases of suspected organophosphorus and carbamate pesticide poisonings in wildlife, including birds, were submitted to the Toxicology Laboratory (ARC-OVI) for analysis. A positive diagnosis was made in 135 (39%) of these cases. The majority of cases were from birds, which included Cape vultures (*Gyps coprotheres*) and African white-backed vultures (*Gyps africanus*) and bateleur eagles (*Terathopius ecaudatus*). In one incident 49 vultures were killed when a farmer intentionally laced carcasses with carbofuran in an attempt to control jackal predation. There were 22 incidents of poisoning in helmeted guineafowl (*Numida meleagris*). On nine different occasions blue cranes (*Anthropoides paradiseus*) were poisoned, in one incident 14 birds were reported to have been killed. Over the period of investigation, there were 20 cases of poisoning involving mammalian species, the majority being vervet monkeys (*Chlorocebus pygerythrus*). The carbamate pesticides were responsible for 57 incidents of poisoning. Aldicarb, carbofuran and methomyl were detected in 26, 18 and 12 cases respectively. The majority of organophosphorus pesticide poisonings were caused by diazinon (*n* = 19), monocrotophos (*n* = 13) and methamidophos (*n* = 10).

## Introduction

The organophosphorus and carbamate pesticides are a large group of organic compounds that are used to control or destroy insects, arachnids and nematodes. A large variation in toxicity occurs between individual compounds. The products registered in veterinary medicine are generally safer; however, there are very toxic agricultural organophosphorus and carbamate pesticides used to protect crops. These compounds inhibit acetylcholinesterase with consequent accumulation of acetylcholine at the autonomic ganglia, effector and neuromuscular junctions and central nervous system (CNS) (Osweiler 1996).

Poisoning of wildlife, including birds, is often a highly emotive issue and usually receives considerable media attention. Although there are frequent allegations of intoxication, analytical confirmation of the specific compound(s) involved is lacking. The Toxicology Laboratory of the Agricultural Research Council – Onderstepoort Veterinary Institute (ARC-OVI), in South Africa, receives specimens for toxicological analysis daily. The aim of the laboratory is to render a speedy and reliable diagnostic service to confirm intoxications. The laboratory analyses samples for a range of potential intoxications that occur frequently in domesticated animals and wildlife in this country and in neighbouring states.

## Materials and methods

The Toxicology Laboratory (ARC-OVI) performed all the analyses of suspected pesticide poisonings of wildlife reported in this article. All the specimens (i.e. contents of the upper digestive tract and/or tissue as well as samples from any possible source of the suspected poison) submitted from an alleged incident of poisoning were recorded as a single case. The samples can originate from an individual animal, but frequently more than one animal and sometimes multiple species may be involved. The specific genus and species names were not always provided by the person submitting the samples. If only the suspected bait or poisoned water or feed was received, it was also recorded as a case of poisoning. Pesticide analysis was performed following a standard protocol (SOP). Briefly, the sample was received and registered. A 30 g aliquot of the sample was extracted by shaking the sample in 100 mL ethyl acetate for one hour. Following filtration over anhydrous sodium sulphate, the sample was screened using a gas chromatograph-mass spectrometer (GC-MS) (Saturn 2100T, Varian) fitted with a capillary column (J&W DB–5 column; 30 m × 0.25 mm id. × 0.25 μm film thickness). When necessary, the sample was concentrated by rotary evaporation (Buchi Rotavapor R–124). Water samples (1–4 L) were extracted in two phases. For the first extraction 200 mL of acetone:hexane (4:96) was used. The second phase of the extraction was performed with 100 mL dichloromethane. Both phases were concentrated before screening on the GC-MS.

## Results

During the six-year period (from January 2009 to December 2014), specimens collected from 344 cases of suspected organophosphorus and carbamate pesticide poisonings in wildlife, including birds, were submitted to the Toxicology Laboratory (ARC-OVI) for analysis. A positive diagnosis was made in 135 (39%) of these cases (Table 1). In Table 2 the specific compounds implicated are listed.

**TABLE 1:** Number of confirmed organophosphorus and carbamate pesticide poisonings in wildlife. Analysis performed by the Toxicology Laboratory, ARC-OVI (2009–2014).

Year	Total number of cases/year†	Number of confirmed cases	Guineafowl	Vultures/Raptors‡	Blue cranes	Other bird species‡	Vervet monkeys	Other mammalian species‡	Bait/water/foodstuffs
2009	60	36	7	4	2	9	1	3 (rhinoceros, leopard, lion)	10
2010	65	35	8	3	2	11	6	2 (hyena, lion)	3
2011	51	18	4	2	1	6	1	1 (leopard)	3
2012	47	13	0	2	3	6	0	0	2
2013	59	14	1	4	0	3	0	2 (nyala, jackal)	4
2014	62	19	2	2	1	6	2	2 (jackal)	4
**Total number of cases**	**344**	**135**	**22**	**17**	**9**	**41**	**10**	**10**	**26**

† Confirmed and negative cases

‡ Genus and species names not provided.

**TABLE 2:** Specific pesticides incriminated in incidents of wildlife poisoning in South Africa.

Chemical group	Compound	Number of cases
Carbamate	Aldicarb	26
	Carbofuran	18
	Methomyl	12
	Carbaryl	1
Organophosphorus	Diazinon	19
	Monocrotophos	13
	Methamidophos	10
	Fenamiphos	9
	Fenthion	7
	Chlorpyrifos	5
	Terbufos	4
	Dichlorvos	3
	Dimethoate	3
	Mevinphos	2
	Parathion	2
	Sulfotepp	1
**Total**		**135**

## Discussion

During the reporting period, a number of vultures were poisoned with organophosphorus or carbamate compounds. On a number of occasions only the crops or the digestive tract were submitted and the specific vulture species involved was not provided, nevertheless, the majority of incidents included African white-backed vultures (*Gyps africanus*). The indiscriminate use of highly toxic agricultural pesticides by farmers to kill jackals and feral marauding dogs may have a serious ecological impact, as can be seen from the incident in 2013 in which 49 vultures (48 Cape vultures [*G. coprotheres*] and one African white-backed vulture) were poisoned when a farmer laced sheep carcases with carbofuran to kill jackals preying on new-born lambs (Wildlife Extra.com 2013). This is in contravention of the law as these compounds should be used strictly for the indications for which they are registered (Act 36 of 1947; Government Gazette Regulation No. R.1716 1991). Virani *et al*. (2011) surmised that pesticide poisoning may also play a role in the decline of the vulture populations in East Africa. They even recommended the banning of carbamate pesticides as an important action to be taken to conserve vulture species in the region. In addition, vultures are poisoned for the harvesting of their body parts for trade in traditional medicine (*muthi*) (Botha, Ogada & Virani 2012). Vultures were mainly poisoned with carbofuran (six cases), aldicarb (three cases) and methamidophos (three cases).

There were also two cases where bateleur eagles (*Terathopius ecaudatus*) were poisoned with aldicarb; in one incident five birds were killed. As these eagles are also scavengers they will easily be poisoned when bait appears to be carrion. Another troublesome statistic is that blue cranes (*Anthropoides paradiseus*), South Africa’s national bird, were also deliberately or incidentally poisoned. In total nine cases were reported over the period of research and in eight instances diazinon was detected. In the most severe incident 14 birds died.

Over the six-year period a large proportion of poisoning incidents in birds occurred in helmeted guineafowl (*Numida meleagris*) (*n* = 22). Guineafowl are illegally harvested by indigent persons, who, after removing the intestines, consume the carcase. This practice used to be a common occurrence, in which maize kernels were soaked in monocrotophos and placed in the vicinity of a water source, but since the banning of monocrotophos in 2005 (Van Zyl 2013) this practice is reported less frequently. During the period of investigation monocrotophos caused the largest number of incidents involving guineafowl (five cases), followed by four cases of fenamiphos and three cases of methomyl poisoning. A number of other bird species, such as pigeons, doves, hornbills, egrets and sparrows, were also poisoned, totalling 41 cases (Table 1).

Twenty cases of pesticide poisoning in mammals were confirmed, including vervet monkeys (*Chlorocebus pygerythrus*), lions (*Panthera leo*), leopards (*Panthera pardus*), a hyena, a rhinoceros, an nyala (*Tragelaphus angasii*) and jackals. Vervet monkeys are sometimes considered to be a nuisance and ten incidents of aldicarb and carbofuran (five cases each) were recorded. The lions (two cases) were killed with aldicarb and terbufos; the leopards (two cases) with carbofuran and aldicarb; the hyena (one case) with terbufos; the rhinoceros (one case) with carbofuran; the nyala (one case) with monocrotophos and the jackals (three cases) with carbofuran and methomyl (Table 1).

The previous report emanating from the Toxicology Laboratory ARC-OVI was published 20 years ago (Fourie *et al*. 1996). The authors ascribed 134 cases of poisoning to acetylcholinesterase inhibitors over a seven–year period (1988–1994). In the current six-year review (2009–2014), 135 cases of poisoning involving these pesticides were recorded. In the earlier report by Fourie *et al*. (1996) the highest number of cases were the result of monocrotophos (*n* = 72), followed by diazinon (*n* = 11) and parathion (*n* = 10) poisoning, all organophosphorus compounds. This current report indicates that the carbamate pesticides (aldicarb, carbofuran and methomyl) were used in the majority of intoxications in wildlife (*n* = 56) or 40% of all occurrences.

**FIGURE 1:**
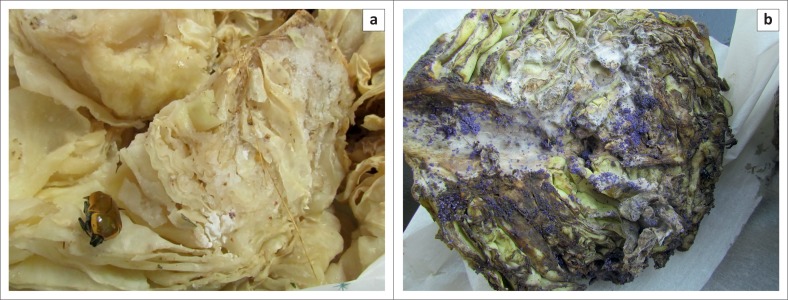
Cabbage laced with (a) methomyl (white powdery substance) and (b) carbofuran (purple coloured granules). Cabbages were placed out most probably in an attempt to poach rhinoceroses.

**FIGURE 2:**
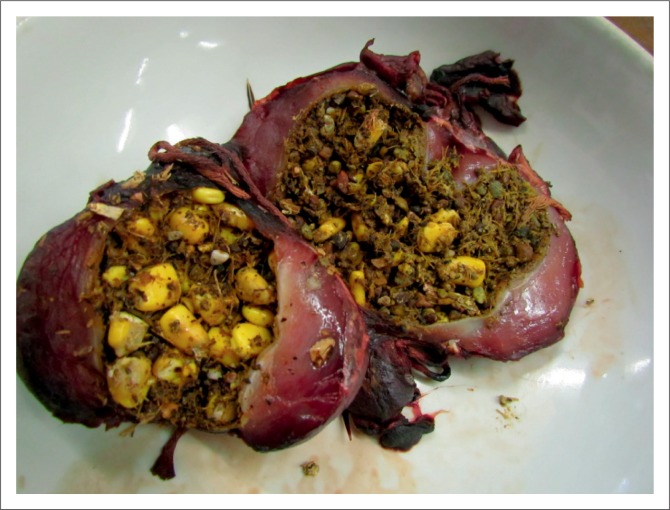
Maize kernels present in the gizzards of poisoned blue cranes. GC-MS analysis of the content revealed diazinon.

**FIGURE 3:**
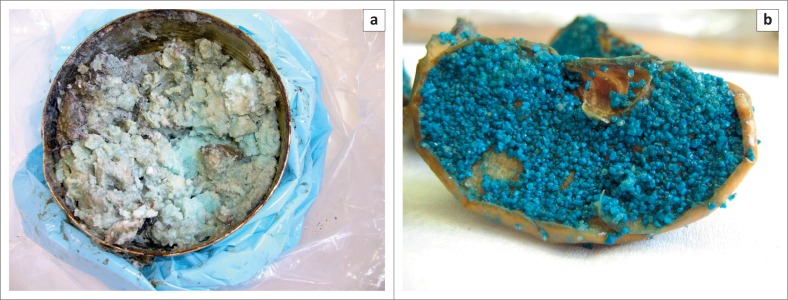
Baits placed out to control vervet monkeys. (a) Carbofuran added to maize porridge (light blue discolouration) and (b) carbofuran-containing granules adhering to apple slices.

The placement of methomyl, a highly toxic carbamate, in cabbages (Figure 1a) was recorded on two separate occasions. Recently, in January 2015, a hitherto unknown purple granular formulation containing carbofuran was used to lace a cabbage (Figure 1b). These incidents were most probably attempts to poach rhinoceroses with the intention of removing the horn. Furthermore, aldicarb and dimethoate, when located, were seized when they were suspected of being used to poach rhinoceroses. Other baits submitted included various potential foodstuffs such as maize kernels (Figure 2) and maize porridge (Figure 3a), apple slices (Figure 3b), oranges and additional feed samples. Some of the pesticides recorded in the report are commercially available as granular formulations (e.g. carbofuran, terbufos and fenamiphos), thus, they are easily subdivided and transferred to smaller containers to be used as baits.

The carbamate pesticides were responsible for 57 incidents of poisoning (Table 2). Aldicarb, carbofuran and methomyl were detected in 26, 18 and 12 cases respectively, and carbaryl was detected in one case. The majority of organophosphorus pesticide poisonings were attributed to diazinon (*n* = 19), monocrotophos (*n* = 13) and methamidophos (*n* = 10).

Aldicarb was voluntarily withdrawn from the market by the company in 2012. A notice for the prohibition of import, export, possession, acquisition, sale, use and disposal of aldicarb and methamidophos was published in the Government Gazette in November 2013. Other pesticides listed in Table 2 that have been banned, withdrawn or are only available for restricted use since 2009 include chlorpyrifos (banned from use in households, home gardens and all domestic uses) and parathion (withdrawn from use on certain crops, ornamental plants as well as for the control of short-horned grasshoppers in 1992 and 1993) (Van Zyl 2013).

As there is no specific pathology associated with acute pesticide poisoning, a diagnosis is reached when analytical data support the history provided and clinical signs described in each case. It is imperative that the correct samples are collected and submitted for analysis. Toxicology laboratories are too often blamed for not being able to isolate a specific toxin when the investigator failed to collect the correct samples. Samples collected during the *post mortem* examination (e.g. contents of the upper digestive tract) as well as samples from any possible source of the suspected poison should be carefully identified and submitted to a diagnostic Toxicology Laboratory. A cover letter providing detailed information should accompany the submission. Biological samples can decay and should either be submitted frozen or on ice (use ice bricks) as soon as possible following the incident. A reliable courier service should be used to deliver the samples timeously.

## Conclusion

The number of cases recorded by the Toxicology Laboratory, ARC-OVI is only the proverbial ‘tip of the iceberg’. Not all incidents of poisoning in wildlife species are reported and samples are not always submitted for analysis to this laboratory, as samples are also sent to other analytical laboratories. To prevent the intentional and malicious poisoning of South Africa’s wildlife species these highly toxic pesticides should be strictly controlled and the acts regulating their use should be enforced stringently. Companies retailing these hazardous chemicals should accept stewardship for their product to safeguard our wildlife, an extremely valuable resource for South Africa.

## References

[CIT0001] Botha A.J., Ogada D.L. & Virani M.Z. (eds.), 2012, *Proceedings of the Pan-African Vulture Summit*, 16–20 April 2012, Masai Mara, viewed 06 July 2015, from https://www.peregrinefund.org/docs/pdf/research-library/2012/2012-PAVS-Proceedings.pdf

[CIT0002] Fourie N., Basson A.T., Basson K.M., Ferreira G.C.H., van den Berg H., Smith J.C.S. et al., 1996, ‘Poisoning of wildlife in South Africa’, *Journal of the South African Veterinary Association* 67, 74–76. 8765066

[CIT0003] Osweiler G.D., 1996, *Toxicology*, Williams & Wilkins, Philadelphia.

[CIT0004] Republic of South Africa, 1947, *Fertilizers, Farm Feeds, Agricultural Remedies and Stock Remedies Act* (Act No. 36 of 1947, 1947), Government Printers, Pretoria.

[CIT0005] Republic of South Africa, 1991, Government Gazette Regulation No. R.1716 of 26 July 1991.

[CIT0006] Republic of South Africa, 2013, Government Gazette No. 37037 of 22 November 2013, Notice 1116 of 2013.

[CIT0007] Van Zyl K., 2013, *A guide to crop pest management in SA – A compendium of acaricides, insecticides, nematicides, molluscicides, avicides and rodenticides*, AVCASA, Pretoria.

[CIT0008] Virani M.Z., Kendall C., Njoroge P. & Thomsett S., 2011, ‘Major declines in the abundance of vultures and other scavenging raptors in and around the Masai Mara ecosystem, Kenya’, *Biological Conservation* 144, 746–752. 10.1016/j.biocon.2010.10.024

[CIT0009] Wildlife Extra.com, 2013, *Mass poisoning of vultures in KwaZulu-Natal*, viewed 02 July 2015, from http://www.wildlifeextra.com/go/news/carbofuran-vultures.html#cr

